# Guidance on the Ethical Use of Mandates for Vaccination Prior to Organ Transplantation in Children: An Analysis From the IPTA Ethics Committee

**DOI:** 10.1111/petr.70488

**Published:** 2026-10-31

**Authors:** M. Damian, M. A. Freeman, S. Rao, A. Wightman, T. Blydt‐Hansen, the IPTA Ethics Committee

**Affiliations:** ^1^ Department of Pediatrics Children's Healthcare of Atlanta, Emory University School of Medicine Atlanta Georgia USA; ^2^ Department of Pediatrics Penn State Health Golisano Children's Hospital, Penn State College of Medicine Hershey Pennsylvania USA; ^3^ Department of Humanities Penn State Health Golisano Children's Hospital, Penn State College of Medicine Hershey Pennsylvania USA; ^4^ Department of Pediatric Surgery Narayana Health Hospitals Bengaluru India; ^5^ Department of Pediatrics, Seattle Children's Research Institute, Treuman Katz Center for Pediatric Bioethics and Palliative Care University of Washington School of Medicine Seattle Washington USA; ^6^ Department of Pediatrics University of British Columbia Vancouver British Columbia Canada

## Abstract

**Background:**

IPTA is a multidisciplinary international professional organization composed of pediatric transplant experts who aim to improve the health of all children with end‐stage organ failure by advancing the science and practice of pediatric transplantation worldwide. The IPTA Ethics committee seeks to provide guidance on ethical issues pertaining to pediatric transplantation in a global context. There is broad consensus that pre‐transplant vaccination decreases the risk of many preventable diseases and the IPTA leadership, committees, and membership unequivocally support immunizations for all children. However, the use of mandated vaccination as criteria for transplant eligibility in the under‐vaccinated pediatric population remains ethically complex.

**Methods:**

In this guidance document, we explore ethical arguments for and against the use of mandated vaccination as a requirement for transplant eligibility.

**Results:**

Considerations in this ethical analysis included assessments of the potential benefits and/or harms experienced by potential recipients for whom vaccination may be required, concerns for appropriate respect for and the limits of parental authority, and societal considerations including organ stewardship, the social contract implicit in organ allocation practices, broader considerations of population safety, and disparities in opportunities for transplant.

**Conclusion:**

This analysis is designed to support clinicians, transplant programs, and, where applicable, regional and national policy makers in evaluating the ethical justifiability of pre‐transplant vaccination requirements for pediatric solid organ transplant candidates. It does not prescribe a single policy approach for all settings but rather offers a structured ethical framework and consensus statements to inform local, regional, and national deliberation.

## Graphical abstract

IPTA strongly supports vaccination for pediatric transplant candidates, but the appropriate use of vaccine mandates remains ethically complex. Policy decisions should be transparent, evidence‐based, equitable, and ethically justified.

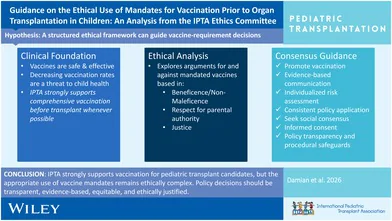

## 1 Introduction: White Paper Development

Under the guidance of the International Pediatric Transplant Association (IPTA) Council, the IPTA committees develop statements expressing the association's key positions and guidance on issues of clinical and ethical importance in pediatric organ transplantation. IPTA is a multidisciplinary international professional organization composed of pediatric transplant experts who aim to improve the health of all children with end‐stage organ failure by advancing the science and practice of pediatric transplantation worldwide.

Immunization against vaccine preventable disease (VPD) is one of the most important interventions available pre‐transplant to mitigate the risk associated with lifelong immunosuppression after a transplant. All of the vaccines provided in preparation for a transplant have been repeatedly proven to be safe and effective. Solid organ transplantation (SOT) is often the best option for children with end‐stage organ failure and ensuring maximum completion of pre‐transplant immunizations is essential to reduce the risk of morbidity or death from VPDs after transplantation.

All transplant programs list a series of recommended and/or required vaccines for transplant candidates, including some specifically made available to people with immune compromise for additional VPD coverage. Pre‐transplant immunization is strongly recommended to obtain the maximum protection from vaccines, since immunosuppression post‐transplant may interfere with the development of optimal immunologic response to vaccinations administered in the post‐transplant setting.

Ethical challenges arise when parents or transplant candidates are vaccine‐hesitant and refuse vaccination before transplant. To address this dilemma, some centers have created policies to mandate that transplant candidates complete all required immunizations before transplant. Among transplant programs, there is substantial variation in how the terms “mandate”, “required” and “recommended” are used to describe criteria for transplant eligibility. For some transplant programs, “mandate” means a strong recommendation, negotiation for counseling or starting immunizations while awaiting organ offers or a plan to verify completion of required vaccines. For others, a mandate is a strict requirement and implies that missing one or more vaccines will make that child ineligible for a transplant until the required vaccines are completed. Similar strict requirements exist for other aspects of pre‐transplant preparation that must be completed before approval, such as review by certain consultants or completion of necessary diagnostic imaging. The degree of stringency with which these “mandates” are developed or applied may vary within the context of public health concerns, such as when the risk of a particular VPD is increased, related to high endemic or epidemic levels of VPD transmission in that community. In general, a vaccine “recommendation” is distinct from a requirement in that it implies parental discretion on whether to proceed.

The recent COVID‐19 pandemic has resulted in increased awareness of the dynamic and evolving nature of some vaccine recommendations and underlying risks of VPD. With this, there has been renewed focus on the ethical questions surrounding requirements for potential transplant recipients to be fully vaccinated before transplantation and what *being fully* vaccinated means [1, 2, 3, 4, 5, 6]. While these and other examinations have been thorough and persuasive in their analysis, it is notable that a unifying consensus on mandates has not yet emerged.

Within this paper the term “mandate” designates a system in which patients with organ failure will not be considered eligible for organ transplantation until they have received all required vaccines. Some vaccines may be recommended (but not required), and patients may proceed to transplant even if they do not receive them. Required vaccines are those deemed most important for protecting recipients after a transplant, such that their completion is verified before going ahead with a transplant. Additionally, for this analysis, we will presume that when vaccinations are mandated, waivers to this requirement will be rare or unavailable to potential organ recipients. While the list of vaccines that are required may vary between programs for reasons of geographic risk, availability, and funding, this variation does not lend itself to a fundamentally different ethical analysis. Therefore, this practice variation will not be addressed in detail.

The IPTA leadership, committees and membership unequivocally support immunizations for all children, including candidates and recipients of organ transplantation. This support is underpinned by extensive scientific evidence that establishes these immunizations as safe and effective for preventing potentially life‐threatening infections. IPTA advocates that all children needing a transplant should have access to and receive all recommended and required vaccines before a transplant, to the fullest extent possible. There is not consensus on whether mandating vaccination before organ transplantation is the ethically optimal approach. Arguments for or against vaccine mandates can be justified within standard ethical frameworks. However, these ethical justifications require careful attention to the strengths and limitations of each argument, as well as thoughtful and explicit statements of the principles that guide the chosen approach. In this context, we have provided a review of arguments both in favor of and in opposition to mandated vaccination of organ transplant candidates, to help guide policy making within institutions and health systems worldwide. We have also included a series of summary statements and recommendations from this analysis for ease of reference.

This document represents the deliberations surrounding this discussion within IPTA. The listed authors represent a variety of clinical disciplines engaged in pediatric solid organ transplantation (Authors MAF, AW, TBH‐Pediatric Nephrology; MD‐ Pediatric Critical Care; SR‐ Abdominal Transplant Surgery; MAF and AW‐Bioethics) The IPTA Ethics Committee further developed this guidance statement, and the IPTA Council has approved it for dissemination and publication. It is intended to support clinicians, transplant programs, and, where applicable, regional and national policy makers in evaluating the ethical justifiability of pre‐transplant vaccination requirements for pediatric solid organ transplant candidates. It does not prescribe a single policy approach for all settings, but rather offers a structured ethical framework and consensus statements to inform local, regional, and national deliberation.

## 2 Background

### 2.1 Vaccines Are Safe and Effective, and IPTA Strongly Supports Comprehensive Vaccination of All Potential Solid Organ Transplant Recipients

Childhood immunizations are recommended worldwide as one of the most successful strategies to improve survival by providing individual and community protection from infectious diseases [7]. Data from the United States (U.S.) Centers for Disease Control and Prevention (CDC) show over 85% reduction in childhood morbidity for 13 of the 16 tracked VPDs, with the remaining all demonstrating over 50% reduction [8]. Similarly, World Health Organization (WHO) data estimate that 3.5–5 million deaths are prevented annually on a global basis from vaccination [7], and routine childhood vaccination has also been shown to result in significant health and economic benefits [9, 10].

The general medical recommendation for pediatric solid organ recipients is to receive all available age‐appropriate vaccines before transplantation. Pretransplant vaccine recommendations mirror local routine immunization practices based on scientific studies showing safety and immunogenicity for specific ages and schedules. In some circumstances, additional vaccines are recommended due to the heightened vulnerabilities of individuals with organ failure or who will receive longitudinal immunosuppression [11]. Maximizing immunizations before transplant is key, as vaccine immunogenicity may be reduced after transplantation [12, 13], and live‐attenuated vaccines are relatively contraindicated for many after transplant [11, 14].

Although several countries have transplant‐related childhood vaccine mandates [15], the specific policies in different jurisdictions depend on politico‐cultural influences, VPD epidemiology, and vaccine availability, cost, and funding. As such, what constitutes “complete” vaccination will differ by country and may also vary between transplant programs in the same region.

### 2.2 Under‐Vaccination Before Solid Organ Transplantation Is Associated With an Increased Risk of Vaccine‐Preventable Disease Compared to Fully Vaccinated Recipients

For children living with a transplant, infection remains one of the most common causes of morbidity and mortality, especially in the first year after transplantation [16, 17]. In one study, 1092 of 6980 (15.6%) SOT recipients experienced 1471 cases of VPD requiring hospitalization [17]. In the 87.9% of these VPD associated hospitalizations that occurred after initial transplantation, 17% of children required intensive care admission, 8% required mechanical ventilation, and 1.3% died [17]. Invasive pneumococcal infection, such as bacteremia and pneumonia, is common (176 episodes per 100 children) after SOT [18], especially in children who are missing the pneumococcal vaccine [19]. Besides infections, SOT recipients are at higher risk for vaccine‐preventable cancers such as cervical cancer and head and neck cancers from human papillomavirus (HPV) [20].

### 2.3 Under‐Vaccination in the Pre‐Transplant Population Is Common and Multifactorial

A 2013 survey of 138 pediatric transplant programs in the U.S. identified that only 12% of programs had written policies regarding under‐vaccination, with only 4% having written policies regarding parental refusal of vaccination [20]. While at most centers, families followed vaccine recommendations, 39% of programs reported having at least one family refusing vaccinations, and 47% of centers had listed an unvaccinated child for transplant [21]. A 2019 study noted that regardless of reason, only 30% of children from 34 pediatric liver transplant centers in North America were up‐to‐date on vaccines before transplant [22]. Feldman et al. [12], found that only 29% of 281 children who underwent SOT between 2004 and 2011 were up to date with the required immunization based on CDC guidelines, and over 51% were missing more than four immunizations. While reports from European countries identify 70%–80% of pediatric transplant candidates as being fully vaccinated per local vaccine schedules before transplant, there is still room for improvement [23, 24].

Under‐vaccination can occur due to a variety of factors. In some circumstances, the acuity of underlying illness with the need for urgent organ transplantation may preclude a family from completing the recommended vaccine schedule before transplantation. Very young patients may require organ transplantation before they would be considered eligible for certain vaccinations. Other medical factors include allergies to vaccine components or contraindication to live vaccines due to ongoing immunosuppression. Some patients may fail to develop laboratory evidence of seroconversion despite receiving a vaccine [25]. Another reason for under‐vaccination is missed opportunities to vaccinate. Many specialty and transplant clinics do not have vaccines on hand, while many pediatricians defer vaccines if a child is ill, further contributing to under‐vaccination.

Vaccine schedules differ worldwide. For example, while the pneumococcal conjugate vaccine is routinely administered in North America, Cuba, Egypt, Czechia, and Ukraine have not included this immunization in their routine schedules [26]. In addition, administration of the human papillomavirus vaccine is not routine in much of Africa, Eastern Europe, and Asia [27]. Even in locations where these vaccines are commercially available, the cost when uninsured may be prohibitive.

In other circumstances, under‐vaccination occurs due to a conscious choice to refuse, delay, or avoid certain vaccinations. This may be influenced by social and economic factors, including access to immunization, resource allocation, or political factors [28, 29]. It may relate to fear from disinformation, or mistrust in authority or government related to historical abuses and community experience [28, 29, 30, 31]. It may also be influenced by low risk‐tolerance for adverse effects with some vaccines (e.g., Guillain‐Barré syndrome or myocarditis), which, although very rare, are quoted as potential risks when some of vaccines are administered [32, 33]. In many countries, parents have the right to refuse vaccination due to religious or personal belief exemptions, and this can play a significant role in their willingness to complete vaccination [34].

While there has been progress on enhancing access to childhood vaccinations globally over the past several decades in regions where vaccines had not been easily available historically, unfortunately this progress has not been uniform [35]. During this time, vaccination rates for several childhood diseases, including measles, have declined in countries where high rates of vaccination were previously achieved [35, 36]. The recent COVID‐19 pandemic was a particularly challenging event, as disruption in routine pediatric care in many countries likely contributed to rates of delayed and under‐vaccinated children in the affected cohort [37]. In the context of social upheaval in the post‐pandemic period, there is evidence that social factors, including political orientation, may be resulting in increased vaccine hesitation, increased concern for side effects, and decreased vaccine confidence in certain populations [38, 39, 40]. Concurrently, measles infections have been increasing, with several countries being at risk for losing or have recently lost measles elimination status [41].

IPTA recommends that clinicians pursue all available strategies to promote vaccination, including those recommended by the World Health Organization and national advisory committees on immunizations [6, 42, 43]. This includes respectful, empathetic engagement with parents to address vaccine concerns. Evidence suggests that clinicians should first use a presumptive communication strategy, in which proceeding with vaccination is stated as the normative and expected outcome of the clinical encounter [43]. This technique is ethically appropriate and consistent with standard frameworks of beneficence provided that there are opportunities to engage in further communication if patients and/or families raise objections or concerns regarding proceeding with immunization.

## 3 Ethical Analysis

There is robust scientific evidence that pediatric organ transplant recipients benefit from the completion of the recommended vaccine schedule before undergoing SOT [17, 44, 45], and as such broadly recommending complete vaccination prior to transplantation is ethically appropriate. There is also robust scientific evidence that in most circumstances of organ failure, SOT would be expected to yield reduced morbidity and mortality as compared to either simple medical management of the sequelae of organ failure or reliance on organ function replacement therapies. However, these facts, in isolation, are insufficient to fully address the question of whether mandating vaccination for solid organ transplant eligibility is an ethically justified response if it denies organ transplant to individuals who are under‐vaccinated. Evaluation of this question also requires careful consideration of the appropriate degree of respect for and limits of parental authority, as well as consideration of the appropriate role and justifications for the authority of transplant programs in determining transplant eligibility.

These questions can be considered by the framework of Principlism, a commonly used method of ethical analysis within medicine [46]. The principles considered with this framework are described in (Table 1).

**TABLE 1:** Overview of principlism.

Principle	Brief description
Beneficence	Health practitioners should seek to provide care that would be expected to do the most good for their patients
Nonmaleficence	Health practitioners should seek to avoid care that would be expected to harm their patients
Respect for autonomy	Health practitioners should provide care consistent with the goals and values of the patient receiving care. This should be guided by the decisions of the informed and competent patient whenever possible
Justice	Health practitioners should seek to ensure that only clinically and ethically relevant facts are used as the basis for medical decision‐making. Within the broader health care system, the principle of justice suggests that the burdens and benefits of medical care be fairly distributed

*Note:* Excerpted with permission from Pediatric Nephrology (2022), Springer International Publishing [47].

It should be noted that while Principlism typically references respect for autonomy within the context of addressing adult patients, respect for parental autonomy and/or authority serves a similar role within the context of ethical assessment in the pediatric setting. While the heading “respect for autonomy” is incorporated in the following analysis due to the broad familiarity of this term, specific limitations of applying this principle in the pediatric setting are addressed in the proposed arguments and counterarguments described.

Given the complexity of the issue, the broader IPTA community has been unable to reach consensus on whether or not vaccine mandates are generally ethically justifiable, especially as any given transplant program may consider different factors to determine eligibility for pediatric organ transplantation. As such, we considered representative arguments for and against mandated vaccination rooted in each of the principles. While individual authors of this paper may find any one of these representative arguments to be more or less persuasive, these arguments as presented are coherent and consistent with standard ethical analysis. They have been included to assist in guiding ongoing discussions and policy development on a local, regional, and national level.

### 3.1 Beneficence

#### 3.1.1 Argument for the Requirement of Mandated Vaccination Before Solid Organ Transplantation

The argument for mandating vaccination before proceeding to SOT is related to the increased risk of VPD morbidity and mortality in the post‐transplant setting. Under a beneficence framework, proponents of vaccine mandates would argue that providing immunity to VPD before causing them to be at increased risk is paramount. Furthermore, a percentage of the vaccine‐hesitant population might proceed with vaccinating their minor children if a mandate was present, and who would not vaccinate absent such a requirement. In support of this argument, when vaccination is mandated as a requirement for school participation, vaccination rates in those communities increase [48, 49]. This is expected to reduce the burden of vaccine‐preventable infectious diseases in the post‐transplant population.

While this is a plausible argument and matches the described experience of many providers, robust evidence is lacking in the published literature that mandating vaccination as a requirement for transplant eligibility results in increased vaccination rates within the under‐vaccinated pediatric organ failure population compared to other less coercive engagement strategies.

#### 3.1.2 Argument Against Mandated Vaccination Before Solid Organ Transplantation

Within a beneficence framework, the argument against a vaccine mandate is based upon acknowledging that organ failure also represents a period of increased morbidity and mortality from infectious causes. As such, this counterargument would posit that the potential survival benefits of organ transplantation related to restoration of organ function may outweigh the associated increased risk of infection from immunosuppression in the under‐vaccinated population. Additionally, the post‐transplant risk from infection differs with each VPD pathogen. In some cases, risk for community transmission is rare when there is otherwise available herd immunity. In other instances, additional mitigation strategies or pre‐emptive therapy may be employed when there is a known potential exposure to limit the severity of infection. As such, the degree of expected benefit/risk mitigation of any vaccine for a given patient is contextual, with varying levels of expected benefit in different circumstances. While a broadly applied mandate for targeted or comprehensive vaccine mandate prior to organ transplantation might provide benefit in some circumstances, the survival benefits of prompt organ transplantation are less contingent on circumstances. Again, it should be noted that while this is a plausible argument, evidence supporting this argument is lacking in the published literature.

### 3.2 Non‐Maleficence

#### 3.2.1 Argument for Mandated Vaccination Before Solid Organ Transplantation

Within a non‐maleficence framework, the argument in favor of mandating vaccination is that following transplantation, under‐vaccinated patients will accrue a greater risk of significant morbidity and mortality from VPD that they would not otherwise face due to the use of immunosuppressant medications. As such, under‐vaccination in this population could be actively harmful and lead to severe illness due to VPDs, including multi‐organ failure and death with severe infection.

#### 3.2.2 Argument Against Mandated Vaccination Before Solid Organ Transplantation

The argument against vaccine mandates before proceeding to SOT within a non‐maleficence framework is that such a mandate might limit or delay access for some patients to organ transplantation, thereby accruing ongoing risk of morbidity and mortality from organ failure that a transplant would avoid. This conflicts with the principle of non‐maleficence. In the case of heart, liver, and lung transplantation, this risk can be reasonably expected to manifest as death from organ failure. While therapies such as chronic hemodialysis or peritoneal dialysis may allow a more extended period of survival in the setting of kidney failure, children receiving chronic dialysis are still at a substantially increased risk of morbidity and mortality as compared to pediatric kidney transplant recipients [50, 51, 52, 53]. Additionally, if vaccines are mandated and proceeding with vaccination is perceived as unreasonably coercive, the mandate may undermine trust, adversely impacting the therapeutic relationship with the transplant team.

From a non‐maleficence perspective, ethical evaluation should focus on the magnitude and nature of post‐transplant clinical risk associated with under‐vaccination, rather than on the moral attribution of responsibility for that under‐vaccination. Clinical risk assessment and judgments about parental decision‐making represent distinct ethical domains and should not be conflated. As an example, the risk of post‐transplant VPD may be similar in a patient who is under‐vaccinated due to patient or family preferences as it would be for patients who are under‐vaccinated due to a medical contraindication (e.g., serious vaccine allergy, ongoing immunosuppression), acuity of organ failure, or who are too young to receive certain vaccines. In such cases, treating people differently *based on a non‐maleficence argument alone* would not be ethically justified. Instead, what matters ethically is the magnitude and nature of the clinical risk associated with under‐vaccination after transplantation, rather than the underlying reason for incomplete vaccination. As an example, a patient who underwent organ transplant prior to being considered eligible for measles vaccination would be expected to have a similar post‐transplant risk as a child who was undervaccinated as a result of parental refusal of measles vaccination. When patients face comparable post‐transplant risks from lacking vaccine‐derived protection, the principle of non‐maleficence cannot justify a decision to treat them differently solely on the basis of whether their under‐vaccination is attributable to medical contraindications, limited access, or parental refusal. This does not mean that evaluation of the reason for under‐vaccination is an inappropriate ethical consideration per se. Instead, any differential treatment of such cases would require justification grounded in other ethical principles, such as justice, rather than in non‐maleficence alone.

### 3.3 Respect for Autonomy/Respect for Parental Authority

#### 3.3.1 Argument for Mandated Vaccination Before Solid Organ Transplantation

Proponents of vaccine mandates would note that such a mandate does not violate respect for patient autonomy or parental authority. Eligibility for SOT is contingent upon several factors, and respect for autonomy does not mean a program is obliged to change those criteria for patients and families who are under‐vaccinated. For example, a program may require specific evaluations, such as imaging studies or psychosocial assessment, as part of the transplant evaluation and tacit or even explicit agreement to engage in standard post‐transplant care protocols following transplantation. Deferring or forgoing transplant listing because that testing is not completed is not typically considered a challenge to parental authority. In this structure, vaccine requirements are simply one of many requirements necessary for transplant eligibility as established by a transplant program. They should not be considered a distinct or more substantial challenge to parental authority or patient autonomy than other requirements.

#### 3.3.2 Argument Against Mandated Vaccination Before Solid Organ Transplantation

Opponents of mandated vaccination would note that, due to the vital health benefits of organ transplantation, withholding transplantation when under‐vaccination is specifically related to the expression of parental autonomy runs the risk of being perceived as coercive and undermining autonomous patient and/or family decisions. Moreover, given that children may lack the capacity or agency to override parental decision‐making, the affected child has a limited ability to act in their own best interest autonomously. Although the adjudication of transplant ineligibility related to under‐vaccination results in that case from parental decisions, it is the child who will bear the health consequences of forgoing organ transplantation.

### 3.4 Justice

#### 3.4.1 Arguments for Mandated Vaccination Before Solid Organ Transplantation

##### 3.4.1.1 The Duty of Organ Stewardship

If all factors are otherwise equal, there is an expectation that the under‐vaccinated population will accrue an increased risk of morbidity, organ failure, or death due to VPD as compared to those who are more fully vaccinated. Therefore, proceeding to organ transplantation in this group would represent an unjustified use of donor organs, which can be broadly considered a scarce societal resource. This can be regarded as harmful to the broader society, since optimizing the use of donated organs for allograft and patient survival is a priority of organ allocation systems. This duty of “Organ Stewardship” is overtly manifest as government structures and processes in some jurisdictions that evaluate transplant center patient outcomes as a criterion for participation (and funding) in the organ transplantation systems. While the increase in absolute risk of allograft loss or patient mortality from VPD in under‐vaccinated organ transplant recipients is difficult to quantify, avoidance of this relative increased risk would be consistent with prioritizing organ stewardship. Therefore, in this societal context, mandated vaccination as a requirement to be eligible for organ transplantation could be ethically justified.

Similarly, one might consider a recommendation that a patient be vaccinated before transplantation to be akin to recommendations that the patient adhere to other treatment recommendations before transplantation. As such, a refusal to receive required vaccinations is a form of non‐adherence and a potential signal of a risk of decreased adherence to post‐transplant care protocols, including medication regimens and monitoring. Transplanting patients who are at increased risk for poor adherence and, therefore, of associated allograft failure post‐transplant could also violate the transplant program's duty of organ stewardship.

It is important to note that within this argument, the reason that a given transplant candidate is undervaccinated becomes ethically meaningful. If a patient is medically ineligible for vaccination due to factors such as age or allergic response, or if the urgency of organ failure necessitates proceeding to transplant before vaccination can be completed, they are not “non‐adherent” to treatment recommendations or violating program policy; appropriately written policies and careful clinical recommendations acknowledge these clinical limitations. In contrast, a patient or family who refuses required vaccinations would be making an ethically distinct decision to break with recommended clinical care, and this distinction can be ethically relevant from the standpoint of a justice‐based analysis.

##### 3.4.1.2 Transplant Population Safety

The standard justification for mandating vaccination as a requirement to participate in some communal activities, such as school, is that the use of mandates both improves the expected health of the individual child through vaccination and reduces the likelihood that under‐vaccinated individuals will serve as an infection reservoir within that communal space. This increases the risk of infection for other members of that community.

Hospitals and clinics caring for patients with organ dysfunction and organ transplant recipients represent a similar communal space. Due to the organization and structure of these shared clinical spaces (including communal waiting areas, shared examination rooms, geographic or temporal grouping in “transplant” clinics, etc.), under‐vaccinated transplant recipients are likely to encounter other individuals who remain at increased risk for infection due to their immunosuppressed and infection‐vulnerable state. If under‐vaccinated transplant recipients are permitted to be present in those spaces, they could expose other vulnerable individuals to VPD who would thereby experience a preventable harm that violates the principle of justice.

##### 3.4.1.3 Violations of the Social Contract Implicit in Organ Allocation Practices

Under‐vaccination due to volitional decisions in the pre‐transplant setting may represent a fracturing of the social contract that is implicit in organ transplantation, particularly from deceased donor sources. Given that deceased donor organs can be best thought of as a societal resource and given that adherence to medical recommendations is a well‐established criterion for organ transplantation, individuals who seek transplantation without the burden of adhering to all recommendations may be thought to be seeking the ‘payout’ of a societal agreement without paying the ‘price’. This may be interpreted as a violation of the principle of Justice.

#### 3.4.2 Argument Against Mandated Vaccination Before Solid Organ Transplantation

##### 3.4.2.1 In Opposition to the Presented Arguments for ‘Organ Stewardship’

###### 3.4.2.1.1 Addressing the Question of Under‐Vaccination as a Preventable Cause of Poor Transplant Outcome

Although it is true that infection with a VPD could ultimately affect allograft function and survival, it is essential to note that all transplant recipients experience different levels of risk for post‐transplant morbidity and mortality due to other clinical considerations such as age, donor organ qualities, primary disease recurrence risk, comorbid health conditions, and severity of illness prior transplant. Such other considerations do not affect transplant eligibility unless their persistence predicts that post‐transplant morbidity and mortality will be excessively high. Moreover, most formal allocation schemas explicitly reject efforts to focus primarily on optimizing transplant outcomes to ensure that allocation achieves fairness and avoids introducing systemic biases. Optimization of outcomes is a concurrent goal of an allocation system, but is weighted so that fairness in allocation is not systematically jeopardized. In this context, the duty of organ stewardship may be better understood as the need to identify patients who *could benefit* from transplant (i.e., setting a threshold of acceptable absolute risk of poor transplant outcome) rather than identifying and prioritizing patient who *will benefit the most* (i.e., focusing on the relative risk of poor transplant outcome for one potential candidate as compared to another).

###### 3.4.2.1.2 Addressing Questions of Poor Adherence to Recommended Vaccination as a Determinant of Post‐Transplant Adherence

While it may be assumed that under‐vaccinated patients and families may be less likely to adhere to other medical recommendations, empiric evidence linking vaccine hesitancy more generally with medical nonadherence is lacking. However, there is evidence that non‐adherence in general may be associated with other social and economic determinants of health and that providers may have biases that influence their perception of which potential candidates are ‘non‐adherent’ [54, 55]. As such, a presumption that under‐vaccination is a marker of broad non‐adherence may adversely influence immediate care decisions, and it may also introduce systemic bias that negatively impacts some disadvantaged families and patients within the transplant allocation system.

##### 3.4.2.2 In Opposition to the Presented Arguments for ‘Transplant Population Safety’

Although pediatric transplant recipients may be at increased risk of infectious diseases broadly, it is not clear that allowing under‐vaccinated patients to undergo organ transplantation meaningfully increases the risk of VPD transmission to other organ transplant recipients. In typical (i.e., non‐epidemic) circumstances, the potential risk of VPD exposure and infection to the vaccinated but immunosuppressed post‐transplant population as a result of the presence of asymptomatic, under‐vaccinated peers in the clinical setting is likely not significantly greater than their risk from exposure in other communal spaces. Furthermore, no similar mandates restrict access by parents or siblings to the same clinic spaces, based on their vaccination status, so mandating vaccination prior to organ transplantation does not actually eliminate the risk of co‐mingling of the vaccinated and under‐vaccinated population in the transplant clinic setting. Instead, this risk is mitigated through the standard application of other infection control precautions, such as designated waiting rooms, masking (or excluding) people with respiratory infections, cleaning procedures, health care staff vaccination and adherence to universal precautions. Additional measures may be required during epidemics when a pathogen is highly prevalent within a community, and infection control measures are implemented for all people attending a hospital or clinic to protect other patients and hospital staff. However, under more normal circumstances, attention to these system‐level procedures is expected to have the greatest impact in reducing risk of exposure to VPD for other patients in clinic, which is accomplished without the need to mandate exclusion of those who are under‐vaccinated from transplantation.

More broadly, a significant goal of transplantation is to enable a return to everyday activities. As such, we do not exclude post‐transplant patients from environments in which a “typical” amount of risk is present such as schools and community events. Absent significant evidence that potentially sharing a clinical space with undervaccinated organ transplant recipients represents a meaningful increase in VPD risk in comparison to these everyday activities, a robust argument for mandatory vaccination based on this concern is challenging.

We would note that these suppositions do not preclude the application of a justice‐based argument around an understanding of transplant population safety entirely. Nevertheless, a realistic understanding of the degree of accrued risk is essential for determining the relative strength of the argument.

##### 3.4.2.3 In Opposition to the Presumed Social Contract Implicit in Organ Allocation Practices

Transplant eligibility is typically evaluated using criteria established by the evaluating program, informed by best practices and adhering to local regulations. These criteria consider whether an acceptable patient risk or benefit adheres to a societal understanding of just allocation. The criteria for transplant eligibility set forth by any individual transplant program represent a form of ‘social contract,’ with all the ethical weight implied by that construct. While individual transplant programs should be presumed to make these determinations in good faith and with attention to the overall goals set forth by society, potential transplantation recipients (as well as the population at large) may have only indirect influence on establishing these criteria. Additionally, there may be some practice variation between transplant programs within the same regulatory jurisdictions, which suggests that broad social consensus on what the requirements for organ transplant candidacy may be lacking. Public engagement on questions of organ allocation policy to authentically give voice to various stakeholders would be instructive but is not always sought. The consensus may also differ based on social norms in multiple jurisdictions. An assumption that there is a social expectation that patients who are under‐vaccinated should be excluded from transplant eligibility is not well tested, and without a public consensus, should not serve as justification for this practice within a justice framework.

Finally, it should be noted that variation in transplant eligibility among different programs raises other justice‐related concerns. It may privilege some organ transplant candidates with the resources to seek evaluation in multiple programs, with differing immunization criteria for candidacy. When potential candidates are situated differently within a system due to characteristics that should not be ethically relevant (i.e., wealth, ability to travel, proximity to several transplant programs), this introduces an additional conflict with the principle of justice.

## 4 Summary Statements and Recommendations

Based on examination and discussion of these arguments, the IPTA Ethics Committees has reached consensus on the following statements and recommendations. These statements are also summarized in (Table 2), with an accompanying explanation:

1. IPTA strongly recommends that children be vaccinated, optimally prior to SOT. This recommendation is underpinned by extensive scientific evidence to establish that these immunizations are safe and effective for preventing potentially life‐threatening infections after solid organ transplantation. Transplant programs should support access to all required vaccinations as part of the pre‐transplant evaluation process, to the fullest extent possible.
2. In the setting of disinformation or mistrust, transplant programs should employ evidence‐based approaches for education and communication to support informed decision‐making by patients and facilitate complete vaccination with patients/families and other providers involved in the patient's care. Transplant programs should, in parallel, address misinformation and knowledge gaps of providers to avoid missed opportunities for complete vaccination.
3. Arguments for mandated vaccination based on assessments of beneficence and non‐maleficence are ultimately based on the comparison of the empiric risk of either proceeding to transplantation in the setting of under‐vaccination or remaining under‐vaccinated in the setting of serious organ dysfunction, as well as the likelihood that a vaccine hesitant transplant candidate will elect to undergo vaccination as a result of a mandate being in place. In the absence of specific data on the magnitude of relative risk, these arguments should be used sparingly to establish ethical policy.
4. Vaccine requirements based on assessing potential benefits and/or harm to the individual should be applied uniformly to all transplant candidates. Given that the reason for under‐vaccination does not alter the associated risks and benefits of proceeding to transplantation while under‐vaccinated for a given patient, it is not ethically relevant within arguments focused on overall risk assessment
5. In considering candidacy for each patient, the decision to proceed with a transplant in the setting of incomplete vaccination should be based on empiric assessment of the balance of risk from lacking vaccine‐derived protection for specific vaccines after transplant against the risk from deferral of transplantation in the setting of organ failure.
6. Arguments regarding mandated vaccinations as a requirement for transplant eligibility based on justice considerations are ethically coherent, but should be explicitly addressed as part of policy development. Ideally, vaccination requirements that are more stringent than existing societal standards that are grounded in the principle of Justice should be resolved at the regional/societal level, by relevant governing bodies seeking societal consensus that reflects shared values and priorities
7. To the extent that a national/societal consensus has not yet been established, any program‐level vaccination mandates or requirements that may be imposed should be rooted in the principles of beneficence or non‐maleficence. These policies should be applied uniformly and justified by empirical medical necessity while avoiding values‐based considerations.
8. As with other potential transplant‐related risks after transplantation, patients who may proceed with a transplant in the setting of incomplete vaccination should do so in the setting of fully informed consent regarding such risks, which includes discussion of additional potential measures that may mitigate these risks
9. If transplant programs adopt vaccination requirements, procedural safeguards should accompany such policies. These should include transparent documentation of required vaccines and their justification, clearly defined medical exemptions, and a structured review or appeal process. Such governance mechanisms enhance fairness, accountability, and public trust, particularly in areas where societal consensus remains unsettled.

**TABLE 2:** Summary of recommendations.

Statements and recommendations	Explanation
1. IPTA strongly recommends that children be vaccinated, optimally prior to solid organ transplantation This recommendation is underpinned by extensive scientific evidence to establish that these immunizations are safe and effective for preventing potentially life‐threatening infections after solid organ transplantation. Transplant programs should support access to all required vaccinations as part of the pre‐transplant evaluation process, to the fullest extent possible	There is robust scientific evidence that potential pediatric organ translate candidates are expected to benefit from the completion of the recommended vaccine schedule prior to undergoing solid organ transplantation
2. In the setting of disinformation or mistrust, transplant programs should employ evidence‐based approaches for education and communication to support informed decision‐making by patients and facilitate complete vaccination with patients/families and other providers involved in the patient's care. Transplant programs should in parallel address misinformation and knowledge gaps of providers, to avoid missed opportunities for complete vaccination	IPTA supports clinicians to pursue all available strategies to promote vaccines, including those recommended by the WHO and other national governing bodies. This includes respectful, empathetic engagement with parents to address vaccine concerns. Presumptive communication strategies, in which proceeding to vaccination is expected and normative, are ethically appropriate
3. Arguments for mandated vaccination based on assessments of beneficence and non‐maleficence are ultimately based on the comparison of the empiric risk of either proceeding to transplantation in the setting or under‐vaccination or remaining under‐vaccinated in the setting of serious organ dysfunction, as well as the likelihood that a vaccine hesitant transplant candidate will elect to undergo vaccination as a result of a mandate being in place. In the absence of specific data on the magnitude of relative risk, these arguments should be used sparingly to establish ethical policy	The argument for mandating vaccination prior to proceeding to solid organ transplantation using a beneficence/non‐maleficence framework would be at its most forceful if programs could engage in evidence‐driven comparison of the many risks the potential recipient could experience including: The relative and absolute risk of morbidity and mortality associated with VPD in the post‐transplant setting in a fully vaccinated versus undervaccinated state The expected increase in vaccination rates among vaccine hesitant candidates in the setting of a regimen of mandated vaccination versus strong recommendation and appropriate communication The relative and absolute risk of morbidity and mortality associated with VPD in undervaccinated individuals following organ transplantation as compared to those undervaccinated individuals continuing simple medical management of the sequelae of organ failure or longitudinal reliance on organ function replacement therapies Absent robust empirical evidence addressing these considerations, transplant programs are reliant on the expert opinions of program leadership
4. Vaccine requirements based on assessing potential benefits and/or harm to the individual should be applied uniformly to all transplant candidates. Given that the reason for under‐vaccination does not alter the associated risks and benefits of proceeding to transplantation while under‐vaccinated for a given patient, it is not ethically relevant within arguments focused on overall risk assessment	The risks and benefits of proceeding to transplantation while under‐vaccinated are independent of the cause of under‐vaccination. The uniform application of vaccine requirements based on these risk/benefit assessments serves as a safeguard against bias, inequity and ad hoc decision‐making. While strict vaccine requirements for transplantation may increase vaccination rates among vaccine‐hesitant potential transplant recipients, they may also have the potential to worsen outcomes for those individuals who remain under‐vaccinated and who forgo transplantation
5. In considering candidacy for each patient, the decision to proceed with a transplant in the setting of incomplete vaccination should be based on empiric assessment of the balance of risk from lacking vaccine‐derived protection for specific vaccines after transplant against the risk from deferral of transplantation in the setting of organ failure	Individualized, patient‐centered risk assessment is ethically appropriate when it is structured, evidence‐based, and consistently applied. Such individualization should not result in discretionary or value‐based exceptions but rather reflect clinically relevant variation in urgency, exposure risk, and disease severity. If these frameworks are utilized, careful attention should be paid to ensure that all under‐vaccinated individuals being considered for transplantation are given similar considerations
6. Arguments regarding mandated vaccinations as a requirement for transplant eligibility based on justice considerations are ethically coherent, but should be explicitly addressed as part of policy development. Ideally, vaccination requirements that are more stringent than existing societal standards that are grounded in the principle of Justice should be resolved at the regional/societal level, by relevant governing bodies seeking societal consensus that reflects shared values and priorities.	If vaccine requirements are justified based on population utility frameworks rather than individual assessments of risk and benefit, this should be considered explicitly, with close attention to the potential for coercion and the resultant harms The establishment of individual health center policies regarding vaccine mandates risks unduly elevating the decisions of small groups of policymakers to the level of a societal policy. These approaches disfavor individuals who lack the economic and social resources to seek care in centers with alternative policies, thereby disadvantaging many groups who face other healthcare disparities
7. To the extent that a national/societal consensus has not yet been established, any program‐level vaccination mandates or requirements that may be imposed should be rooted in the principles of beneficence or non‐maleficence. These policies should be uniform in their application and justified based on empirical medical necessity, while avoiding value‐based considerations	While individual transplant programs may be empowered to establish vaccination requirements as a function of health system structure, their role in selecting appropriate candidates is primarily a result of their acknowledgement of their medical knowledge and expertise. As such, the programs determination of the risk and/or benefits of proceeding to transplantation for a given candidate is a reflection of that medical expertise. Questions of justice and the appropriate degree of respect for parental authority are questions of values, for which medical professionals do not necessarily hold special expertise or authority
8. As with other potential transplant‐related risks after transplantation, patients who may proceed with a transplant in the setting of incomplete vaccination should do so in the setting of fully‐informed consent regarding such risks, which includes discussion of additional potential measures that may mitigate these risks	Appropriate counseling and sustained communication around this clinically important issue are essential to facilitate informed patient and family decision making, a vital aspect of respect for patient autonomy and parental authority
9. If transplant programs adopt vaccination requirements, procedural safeguards should accompany such policies. These should include transparent documentation of required vaccines and their justification, clearly defined medical exemptions, and a structured review or appeal process. Such governance mechanisms enhance fairness, accountability, and public trust, particularly in areas where societal consensus remains unsettled	Actions that enhance and sustain a trust‐based relationship between the providers and transplant programs and the patients and families they serve are vital to both enhance the quality of transplant care and transplant community's shared effort to support childhood vaccination more broadly

## Conflicts of Interest

The authors declare no conflicts of interest.

## Data Availability

Data sharing not applicable to this article as no datasets were generated or analysed during the current study.
